# Polymorphism in magic-sized Au_144_(SR)_60_ clusters

**DOI:** 10.1038/ncomms11859

**Published:** 2016-06-14

**Authors:** Kirsten M.Ø. Jensen, Pavol Juhas, Marcus A. Tofanelli, Christine L. Heinecke, Gavin Vaughan, Christopher J. Ackerson, Simon J. L. Billinge

**Affiliations:** 1 Department of Applied Physics and Applied Mathematics, Columbia University, New York, New York 10027, USA; 2 Condensed Matter Physics and Materials Science Department, Brookhaven National Laboratory, Upton, New York 11973, USA; 3 Department of Chemistry, Colorado State University, Fort Collins, Colorado 80523, USA; 4 European Synchrotron Radiation Facility, 38043 Grenoble, France

## Abstract

Ultra-small, magic-sized metal nanoclusters represent an important new class of materials with properties between molecules and particles. However, their small size challenges the conventional methods for structure characterization. Here we present the structure of ultra-stable Au_144_(SR)_60_ magic-sized nanoclusters obtained from atomic pair distribution function analysis of X-ray powder diffraction data. The study reveals structural polymorphism in these archetypal nanoclusters. In addition to confirming the theoretically predicted icosahedral-cored cluster, we also find samples with a truncated decahedral core structure, with some samples exhibiting a coexistence of both cluster structures. Although the clusters are monodisperse in size, structural diversity is apparent. The discovery of polymorphism may open up a new dimension in nanoscale engineering.

## Abstract (web summary)

Gold nanoclusters are important nanomaterials but their structural assignment can be challenging if single crystals can't be grown. Here, the authors use pair distribution function analysis of X-ray powder diffraction data for Au_144_(SR)_60_ nanoclusters, and show that they exhibit polymorphism.

## Graphical abstract

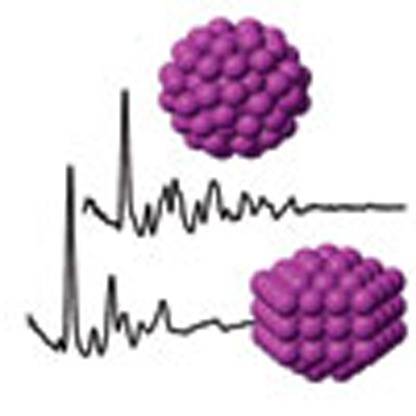

The promise of nanotechnology, to engineer materials at the nanoscale with improved properties, is predicated on the idea that material structure and properties are fundamentally modified on this scale. Gold clusters are prototypical inorganic materials that exemplify this^1^,^2^,^3^,^4^,^5^,^6^,^7^,^8^. In addition to being technologically important in their own right^9^, they are a model system for studying this paradigm, as they form ultra-stable ‘magic number' molecule-like clusters of different sizes^10^,^11^. A major challenge, in the majority of cases where the clusters cannot be crystallized, is to determine their structure. We overcome this ‘nanostructure problem'^12^ by using atomic pair distribution function (PDF) analysis of X-ray diffraction (XRD) data to study the structure of Au_144_(SR)_60_ (where R is the organic part of the thiol), one of the largest of the ultra-stable magic-sized clusters with known composition^13^,^14^. The PDF data successfully yield the core structure, with the surprising result that these clusters exhibit polymorphism. In very recent studies, single crystal structure determination illustrated that the much smaller Au_38_(SR)_24_ interconverts reversibly between two forms, depending on temperature^15^. Here we use PDF to show that polymorphism exists also in the large Au_144_(SR)_60_ cluster, representing the size regime in the transition between clusters forming non-bulk geometric structures and bulk face-centred cubic (fcc) nanoparticles^8^. The discovery of polymorphism brings an additional dimension to the phase space for nanoscale engineering.

The Au_144_(SR)_60_ structure has already been subject to many studies. Initially described as a ubiquitous 29-kDa core-mass compound^14^,^16^,^17^, more recently the composition was determined by mass spectrometry as Au_144_(SR)_60_ (refs 18, 19). Lopez-Acevedo *et al.*^20^ developed a detailed structural model, tested by density functional theory (DFT), where the cluster consists of an icosahedral gold core surrounded by a gold/thiol surface layer. NMR (nuclear magnetic resonance) studies later suggested that all ligands are in symmetry equivalent positions^21^. Scanning transmission electron microscopy (STEM) studies by Bahena *et al.*^22^ were consistent with the icosahedral core and by introducing the NMR symmetry requirement in theoretical calculations they proposed a symmetrized structure model featuring an equivalent ligand arrangement^22^. This model consists of a gold core of 54 atoms arranged as two Mackay icosahedral shells (Fig. 1a), whereas a 60-atom layer covers the 55-site inner core in an ‘anti-Mackay' manner (Fig. 1b). The surface of the cluster structures consist of -SR-Au-SR-type structures (Fig. 1d), referred to as ‘staples'^23^, and in combination this gives the full proposed structure as illustrated in Fig. 1c. The structure is closely related to that of Pd_145_(CO)(PEt_3_) determined by single-crystal XRD^24^.

In this study, we apply atomic PDF analysis to Au_144_(SR)_60_. PDF analysis (described in Supplementary Note 1) has become widely used for nanostructure analysis^25^,^26^,^27^,^28^,^29^ and is a potential tool for nanostructure solution^30^,^31^,^32^. In recent times, PDF has also been applied to the fingerprinting of gold nanocluster structure^33^,^34^. PDF goes beyond conventional X-ray powder diffraction, which typically covers only a narrow range of reciprocal space^35^ and neglects diffuse scattering. The total scattering approach contains significantly more structural information, allowing a quantitative assessment of the structure that is impossible with conventional data from such small particles. We apply PDF nanostructure analysis to Au_144_(SR)_60_ clusters prepared with different ligands (Fig. 1e): phenylethane thiol (PET), *para-*mercaptobenzoic acid (*p*-MBA), butane thiol (SC4), hexanethiol (SC6) and dodecanethiol (SC12). Sample homogeneity is characterized by electrospray ionization–mass spectrometry (ESI–MS) and electrochemical methods. The approach results in full quantitative refinements of the structure of the gold core, with a semi-quantitative assessment of the surface structure. Surprisingly, we find two distinct structural forms for this cluster's core, one based on icosahedra seen in smaller clusters, proposed earlier for this 144 gold atom cluster^20^,^21^,^22^, and one based on close packed decahedra that resemble larger gold clusters and bulk gold. The discovery of polymorphism in gold nanoclusters opens up a new dimension in nanoparticle engineering, presenting the possibility of engineering nanoparticle structure, as well as size and morphology.

## Results

### PDFs from Au_144_(SR)_60_

**Figure 2:**
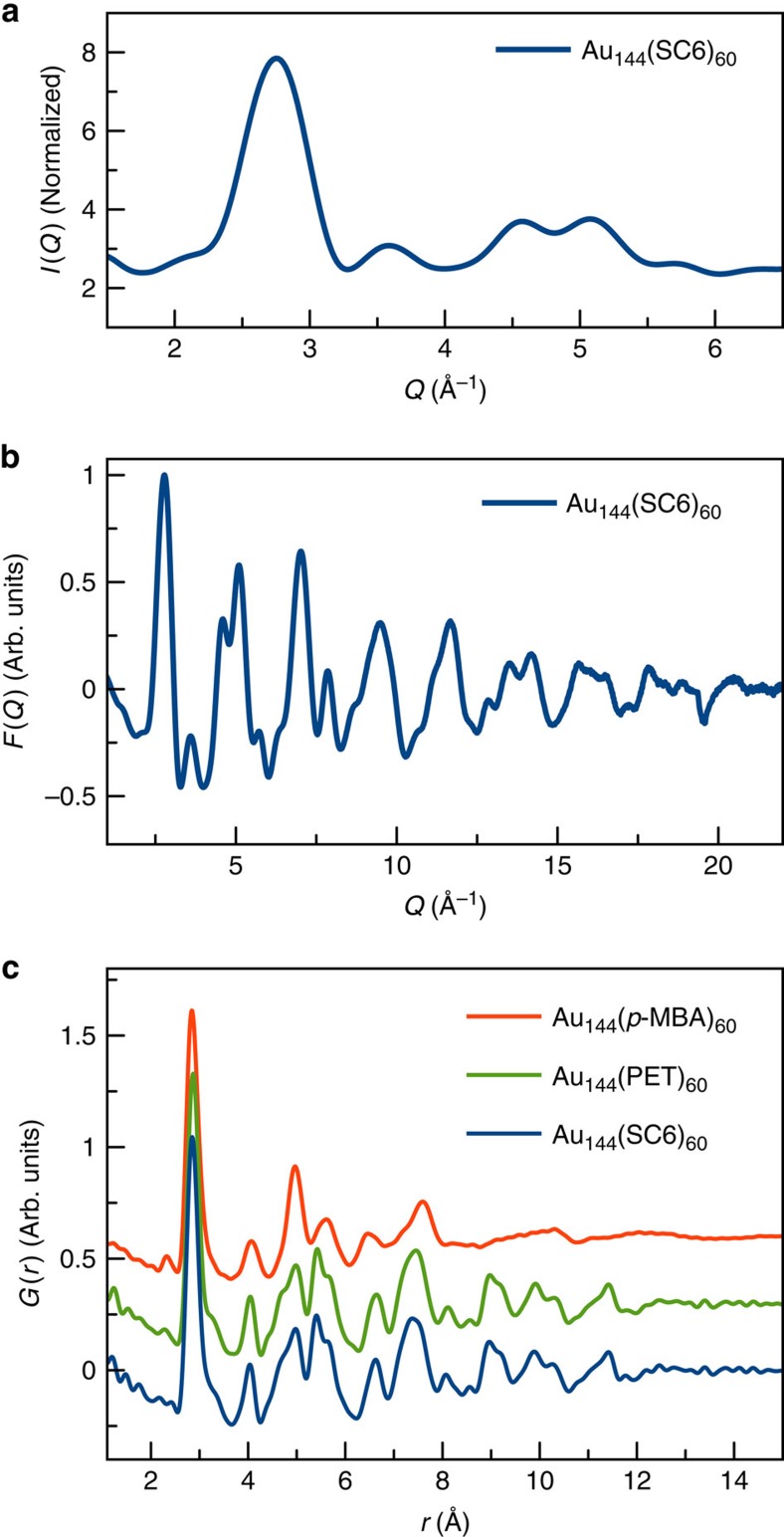
Collected scattering data for Au_144_(SR)_60_ clusters. (**a**) Low *Q* scattering data, corresponding to the conventional XRD signal for the Au_144_(SC6)_60_ sample. (**b**) Total scattering structure function *F*(*Q*) for Au_144_(SC6)_60_. (**c**) PDFs obtained from Au_144_(SC6)_60_, Au_144_(PET)_60_ and Au_144_(*p*-MBA)_60_.

We first investigate the sample prepared with SC6 ligands. ESI–MS data (see Supplementary Fig. 1 and Supplementary Table 1) confirmed homogeneity of this sample, with at least 90% of the sample being Au_144_(SC6)_60_ and a small byproduct (<10%) with ESI–MS peaks, which can be assigned to Au_137_(SR)_56_ (ref. 36). Previously in the literature, this impurity signal has been assigned to Au_144_(SR)_60_ fragments^37^,^38^. No other cluster sizes, such as Au_130_(SR)_50_ or Au_133_(SR)_52_ were detected. The low *Q* scattering signal (where *Q*=4*π*sin(*θ*)/*λ* is the magnitude of the scattering vector), corresponding to conventional XRD data, the total scattering structure function *F(Q)* and the PDF, *G(r)*, from this sample at 100 K are shown in Fig. 2. Owing to the small size of the gold clusters, only very broad scattering peaks are present in the low *Q* signal (Fig. 2a), resulting in too little information to attempt a total structure solution by crystallographic means. In the total scattering structure function *F(Q)* (Fig. 2b) we see that the diffuse scattering extends over a wide range of reciprocal space containing scattering features with rich information that cannot be resolved when just the low *Q* conventional XRD data are used. The PDF, plotted in blue in Fig. 2c, is the Fourier transform of the data in Fig. 2b. This real-space function contains peaks at distances separating pairs of atoms in the structure. The observation of sharp peaks in real-space indicates that the gold clusters have a well-defined structure. The peaks in *G(r)* disappear above 12.5 Å, which puts a lower bound on the diameter of the gold core of the clusters. The first large peak at *ca*. 2.9 Å is the nearest-neighbour gold–gold distance, *r*_nn_, and is sharp. The strength and sharpness of the low *r* peaks suggests a high multiplicity for these distances, indicating a rather well-packed structure. In Fig. 2c, we also show the experimental PDFs from the Au_144_(PET)_60_ sample plotted in green. The similarity of the PDFs from Au_144_(SC6)_60_ and Au_144_(PET)_60_ indicates that these clusters have identical core structures and also establishes the reproducibility of the PDF measurements.

The red line in Fig. 2c shows the PDF from Au_144_(*p-*MBA)_60_. There is a remarkable difference between this PDF and those of Au_144_(SC6)_60_ and Au_144_(PET). These clusters have the same size, as evident from the disappearance of sharp features in the PDF, by the characteristic, well-defined differential pulse voltammetry and from mobility in polyacrylamide gel electrophoresis consistent with Au_144_(SR)_60_. Both the SC6, PET- and *p-*MBA-protected preparations, formed the poorly diffracting hexagonal plate crystals previously observed for these compounds^39^,^40^. The sharp PDF peaks indicate that the Au_144_(*p-*MBA)_60_ clusters also have a well-defined ordered structure. However, their structure is remarkably different from that of the SC6- and PET-terminated clusters: the Au_144_(SR)_60_ clusters are exhibiting polymorphism. We now explore quantitatively the two structural polymorphs, Form I and Form II, of these clusters.

### Form I

**Figure 3:**
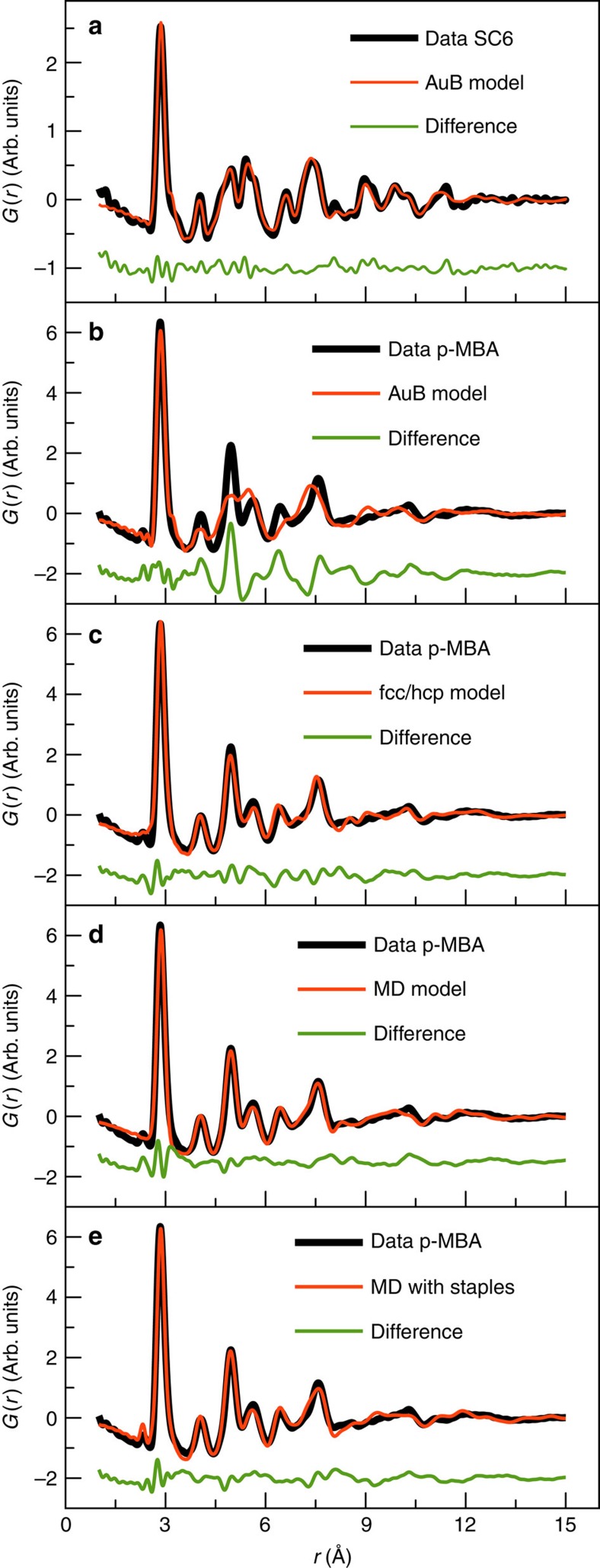
Fits to experimental PDFs. (**a**) Fit of Bahena model to Au_144_(SC6)_60_ data. (**b**) Fit of icosahedral model to Au_144_(*p*-MBA)_60_ data. (**c**) Fit of fcc/hcp model to Au_144_(*p*-MBA)_60_ data. (**d**) Fit of 114 atom decahedral model to Au_144_(*p*-MBA)_60_ data. (**e**) Fit of decahedral model with staples to Au_144_(*p*-MBA)_60_ data.

We begin by calculating PDFs from candidate structures suggested in the literature to compare with the data. The relative atomic positions are highly constrained in the modelling (described in detail in Supplementary Note 2), with only five parameters allowed to vary: a scale factor accounting for the overall PDF intensity, a uniform cluster expansion factor that allows the cluster structure to contract or expand, two isotropic atomic displacement parameter applied separately to the core and surface atoms, as well as a parameter accounting for correlated atomic motion^41^. Therefore, good fits to the data are a strong indicator that the model has captured the correct geometry of the core. To simplify the models, only the Au and S atoms were included in the refinements, as the scattering signal from the organic ligands is negligible (see Supplementary Note 3). Figure 3a shows the calculated PDF from the model suggested by Bahena *et al.*^22^, fitted to the SC6 data^20^. The result of a refinement to the same data, but using the structure reported by Lopez-Acevedo *et al.*^20^ is shown in Supplementary Fig. 2. Both models describe the main features of the PDF of the Au_144_(SC6)_60_ nanoclusters very well, with the Bahena structure giving a slightly better fit to the PDF with agreement factor *R*_W_=16.3%. This confirms the proposed structures of previous theoretical and STEM studies^21^,^22^.

Similar fits to the Au_144_(PET)_60_ sample are given in Supplementary Fig. 3 and Supplementary Table 2, also showing good agreement with the icosahedral model (*R*_W_=15.8%). Furthermore, a direct comparison of the experimental data from the PET and SC6 data show that the two samples give rise to practically identical PDFs as illustrated in Supplementary Fig. 4, where the difference curve between the two PDFs is essentially a flat line. Interestingly, the ESI–MS data indicated ∼16% Au_137_(SR)_56_ in the PET sample, that is, a higher fraction than seen in the SC6 sample. The flat difference curve between the two PDFs would not be expected if the byproduct signal in ESI–MS is coming from a different cluster, that is, Au_137_(SR)_56._ Thus, the PDFs either indicate that the byproduct signal is coming from fragments of Au_144_(SR)_60_ created during the ESI–MS measurement, or that the core structure of Au_137_(SR)_56_ is indistinguishable to that of Au_144_(SR)_60_ Form I. As we see later, the PDF is quite sensitive to small changes in core structure and, although the latter scenario cannot be ruled out, the former is more probable, indicating that our samples are pure Au_144_(SR)_60_. If the latter scenario is correct, it establishes that the core of Au_137_(SR)_56_ is highly similar to that of Au_144_(SR)_60_.

The data shown in Fig. 2 are obtained at 100 K. Scattering data from Au_144_(PET)_60_ were also taken at 300 K, showing no structural changes between the two temperatures (Supplementary Fig. 5). Furthermore, we measured data using three different X-ray energies, ranging from 39 to 87 keV, and all PDFs (Supplementary Fig. 5) showed the same structure.

### Form II

We now turn to the structure of the Au_144_(*p*-MBA)_60_ cluster, which has the very different PDF evident in Fig. 2c. The homogeneity of this cluster sample was characterized by electrochemical measurements. Total scattering data were measured from samples of Au_144_(*p*-MBA)_60_ from two different synthesis batches and as shown in Supplementary Fig. 6 the two PDFs are completely reproduced with a small difference residuum of *R*_W_=7.8%, illustrating reproducibility of the synthesis and reliability of the measurements. We first attempt to use the Form I icosahedral structural model to establish whether this can be made to fit the different PDF by adjusting the refinement parameters. However, the model gives a very poor fit with a large difference between the calculated and measured PDF, and poor fit residuum of *R*_W_=36.0% as shown in Fig. 3b. To further confirm that the sample does not simply contain stable clusters of a different size, for example, Au_102_(SR)_44_ (ref. 3), Au_130_(SR)_50_ (ref. 42) or Au_133_(SR)_52_ (ref. 43), we fitted known structural models for these clusters to the *p*-MBA data. In all cases, the models gave very poor agreements with the data (fits shown in Supplementary Figs 7–9), confirming that the samples are not made up of other stable cluster sizes.

Therefore, other models for the Au_144_(SR)_60_ gold cluster were explored. Initially, we considered only the positions of the 144 gold atoms and ignored the ligands in the model. We based this on the dominating scattering power of gold compared with the thiolates, as discussed further in the Supplementary Note 3. First, a series of close-packed core models were constructed, closely related to bulk fcc gold. These included a 147-atom cuboctahedron, as well as clusters formed by cutting a sphere of ∼144 atoms from fcc and hexagonal close-packed (hcp) lattices. The next attempted model was a two-phase fit of the PDFs from cutouts from fcc and hcp, which has been used as a proxy model in PDF modelling for close-packed structures that contain stacking faults^27^. A summary of these simulations is given in Supplementary Table 3 and Supplementary Note 4. None of the fcc- or hcp-based clusters produced completely convincing fits to the observed PDF. However, the fits were significantly better than for the Bahena model, especially for the fcc/hcp mixture as shown in Fig. 3c. The PDF agreement of the fcc/hcp model was remarkably improved after allowing for a separate expansion ratio for the atoms in the outermost shell, giving *R*_W_=16.3%, a step which was motivated by allowing for a possible surface relaxation. This improved the refinement by fitting the asymmetry in the first Au–Au peak. However, the results indicated that the bond lengths between the atoms in the surface were contracted compared with the bonds in the core and the atomic displacement parameters were excessively large over 0.03 Å^2^ for the core atoms, suggesting the existence of some atomic relaxations that are not part of these simple models. Furthermore, this model contains 141 atoms in the fcc phase and 147 atoms in the hcp phase. We seek a model that can also explain the high stability of the Au core with 144 atoms, whereas spherical chunks of close-packed bulk material would not have special stability. Nonetheless, the fitting results establish that the structure of Au_144_(SR)_60_ is much closer to a three-dimensional close-packed structure than the icosahedral, DFT-derived models.

**Figure 4:**
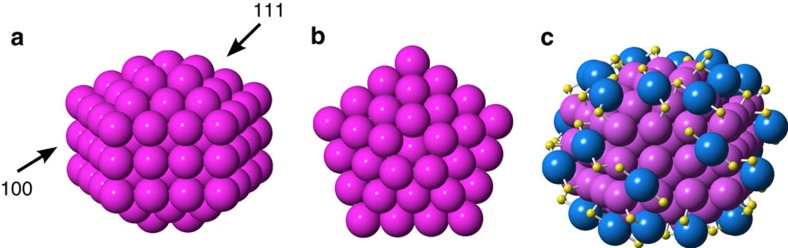
114-Atom and 144-atom ino decahedron cluster. (**a**) Side view. (**b**) Top view. (**c**) Decorated with 60 (SR-Au-SR) staples. Pink spheres show gold atoms in the cluster core, whereas blue spheres show gold in the staple structure. Sulfur is shown in yellow. The organic chains have been left out for clarity.

Our search for close-packed structures that have special atom counts led us to explore a series of Marks decahedral structures that are constructed by introducing twin boundaries along the *(110)* planes of the fcc lattice^44^,^45^, as described in more detail in Supplementary Note 5. Closed shell, truncated decahedra can be constructed with a large range of discrete number of atoms, including 144, that is, the exact number of gold atoms in the cluster. This structure and the fit to the experimental PDF are shown in Supplementary Fig. 10, where excellent fits are seen. However, as described above, thiolate ligands are known to create –SR-Au-SR– or –SR-Au-SR-Au-SR– ‘staples' on gold surfaces^23^,^46^. The short staple, that is, –SR-Au-SR– is mainly seen on larger clusters, where the curvature is small, as would be the case in the ∼2 nm Au_144_(SR)_60_ structure, and Au_144_(SR)_60_ may thus better be represented as Au_114_[(SR)-Au-(SR)]_30_. This pointed us towards a smaller ino decahedral structure as the core of the cluster, as illustrated in Fig. 4a,b. The cluster shown has exactly 114 gold atoms, leaving 30 gold atoms for the staples as required by the putative stoichiometry. The fit of this cluster to the data is shown in Fig. 3d and, as illustrated, the model very well describes the experimental PDF. All distinct sharp peaks up to 8 Å are reproduced and the fit remains very close even at higher *r*-values where the features are broader and less resolved. In studies of smaller clusters, it has been shown that although the core of the cluster is decahedral, a shell of gold atoms may be seen between the core structure and the staple layer^3^. Therefore, we tried stripping down the decahedral structure to a yet smaller core and reattaching the atoms as ‘caps' on the remaining structure^3^. However, interestingly, any modification to the 114 atom decahedral core highly deteriorated the PDF fit, making lower symmetry structures unlikely. This makes us confident in a core structure based on the 114 atom decahedron, closely related to the ino decahedron described by Cleveland *et al.*^17^

Various configurations of the staples on the 114-atom decahedral structure cluster were then considered, where one example is presented in Fig. 4c and other selected models are shown in Supplementary Fig. 11. The process of attaching the staples is described in detail in Supplementary Note 6. Staples were placed on the (*111)* surfaces as previously seen^23^; however, to accommodate all ligands to the structure in a physically sensible manner, staples were also attached to the (*100*) surfaces, although this motif has not yet been reported. Several different models were constructed, which all give comparably good fits to the data with *R*_W_ values of *ca*. 15–18%, with one example shown in Fig. 3e, where the presence of staples fit to the shoulder of the nearest neighbour Au–Au peak. The PDF refinements were somewhat sensitive to the staple attachment, as subtle differences between the features in the fitted PDF can be observed. However, based on PDF data alone we cannot determine the exact ligand arrangement and further studies combining total scattering with techniques sensitive to the ligand attachment are needed to determine the surface structure with full confidence.

Nevertheless, the PDF analysis clearly shows that the Au_144_(*p*-MBA)_60_ core takes a decahedral structure, unlike the Au_144_(SC6)_60_ and Au_144_(PET)_60_ samples described above. We call this second stable structure for Au_144_(SR)_60_ Form II. The decahedral structure fits well in the thiol stabilized gold cluster structure series. From single-crystal XRD of smaller clusters, a strong effect of ligand on internal structure and allowed nuclearity can be inferred, with close-packed and icosahedral structures both observed. For instance, Au_25_(PET)_18_ (ref. 47), Au_38_(PET)_24_ (ref. 48) and Au_133_(SPh-tBu)_52_ (ref. 43) have been determined to have icosahedral cores. This is in contrast to Au_18_(SC_6_H_11_)_14_ (ref. 49), Au_36_(SPh-tBu)_24_ (ref. 50) and Au_102_(*p-*MBA)_44_ (ref. 3), which have close-packed cores. A cuboctahedron-like structure (which also has closed-packed motifs) was seen for the Au_68_(*p*-MBA)_32_ cluster by advanced single-particle electron microscopy methods^51^. It has furthermore been shown that substituting Au by Ag in ligand-free clusters containing *ca.* 312 metal atoms changes the structure from fcc to icosahedral^52^.

### Form I and II coexistence

**Figure 5:**
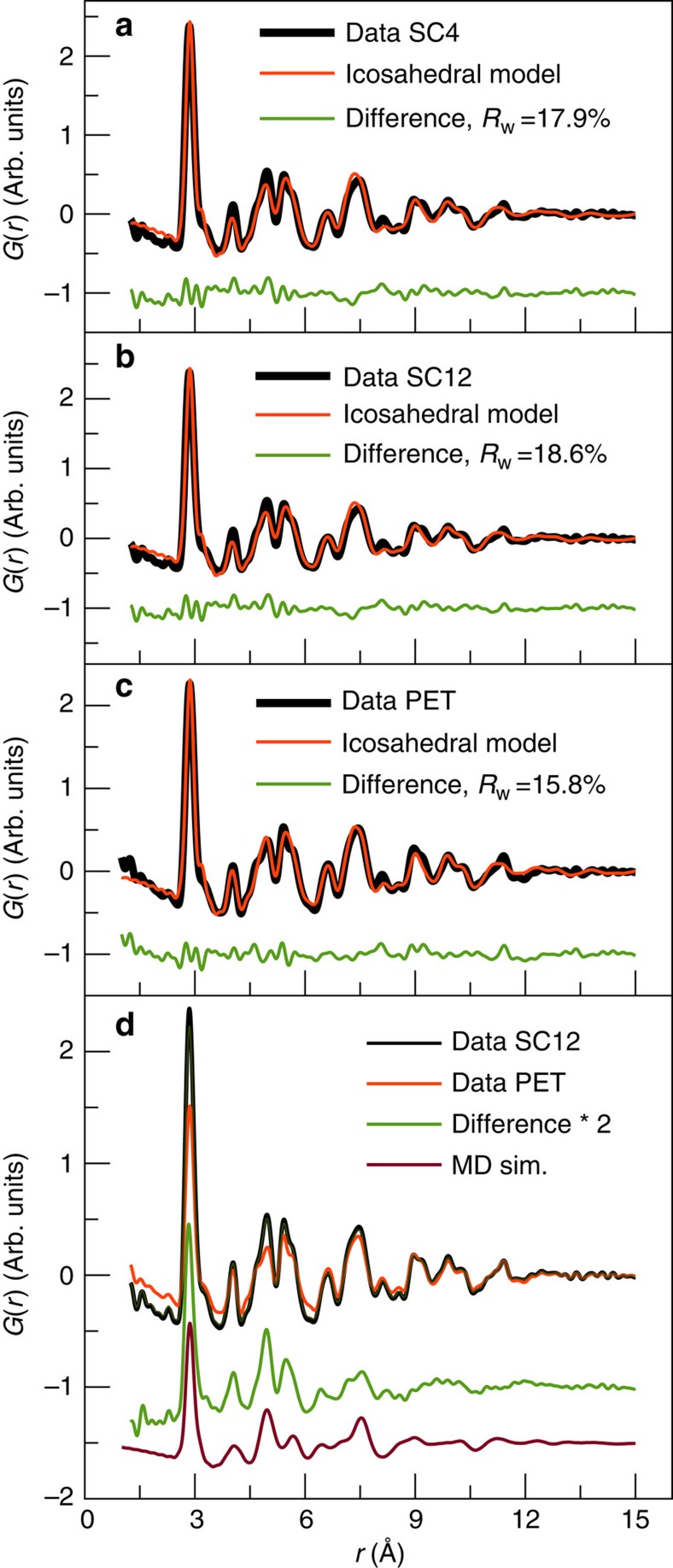
Fits of the Bahena model to experimental data. (**a**) Fit to Au_144_(SC4)_60_ data, (**b**) to Au_144_(SC12)_60_ data and (**c**) to Au_144_(PET)_60_ data. (**d**) Data for the Au_144_(PET)_60_ and Au_144_(SC12)_60_; the difference between them and the calculated PDF from the 114-atom decahedron model. The difference curve has been doubled in scale for clarity.

We next attempted to find a trend in ligand type for stabilizing the different structural forms and tested the effects of using linear thiol ligands of different length, namely SC4 and SC12, which we compare with the SC6 and PET samples. The PDF data for SC4, SC12 and PET are shown in Fig. 5a–c along with fits using the Form I model. The refined parameters are given in Supplementary Table 4, where the data from the hexane thiolated sample (SC6) and PET samples show good agreement with the icosahedral Form I model, and the SC4 and SC12 samples give much larger residuum values of 17.9% and 18.6%, respectively. Interestingly, the disagreement between data and model is particularly large around *r*=5 Å, which is exactly the position for one of the most dominating peaks in the decahedral PDF. ESI–MS data from both the SC4 and SC12 samples showed Au_144_(SR)_60_, as well as impurity peaks corresponding to Au_137_(SR)_56_ in quantities comparable to the PET-protected samples. No other clusters were seen. As the presence of Au_137_(SR)_56_ in the ESI–MS data did not affect the PDF fits to the PET-protected sample and as no other clusters are identified by ESI–MS, we can rule out that the disagreement is due to the presence of other cluster sizes.

In Fig. 5d, the experimentally derived PET PDF (in Form I) has been subtracted from the SC12 calculated PDF and the difference curve is plotted below. Close inspection indicates that it strongly resembles the PDF of the Form II decahedral core structure, as seen when comparing with the calculated PDF from the 114-atom decahedron plotted along with the data. The difference curve has exactly the same features as seen from the decahedron phase, showing that the sample contains clusters of two distinct structures: Form I and Form II. Similar results are seen for the SC4 sample as illustrated in Supplementary Fig. 12. Two-phase fits showed that the SC4 sample contains 12% decahedral clusters (88% icosahedral clusters), whereas the SC12 sample has 14% decahedral clusters, as listed in Supplementary Table 5 and illustrated in Supplementary Fig. 13. When including the decahedral phase in the fit, the resulting *R*-values are reduced to *ca.* 16%. The results unambiguously show that two polymorphs of the Au_144_(SR)_60_ cluster are present.

## Discussion

The question remains which factors affect the polymorph. Previous studies of gold clusters have indicated that ligand length may influence the structure of the gold core^14^. Our total scattering data cannot confirm this trend, as both the longest (SC12) and shortest (SC4) linear ligand give mainly icosahedral clusters, with a smaller fraction of decahedral clusters present in each sample. The fact that we see both the icosahedral and decahedral clusters in samples made with the same ligands illustrate that the structural diversity is not a simple effect of the ligand chain length, bulkiness or bonding strength. It is a clear indication that the two structures are very close in energy. As discussed above, Wong *et al.*^21^ reported ^1^H-NMR studies of Au_144_(*p*-MBA)_60_ clusters, which showed only one doublet in the aromatic region of the spectrum, suggesting that all ligands are in symmetry equivalent positions. Interestingly, ^13^C-NMR studies on 29 kDa gold clusters have shown that the NMR signal is highly dependent on the charge state of the nanocluster. The simple NMR signal indicating symmetry equivalent ligands was seen only when the clusters were in charge state +3, whereas other signals were seen at lower charge states^53^. In recent work, Tlahuice-Floret *et al.*^54^ used DFT to study the effect of charge state on structure of the gold subhalide Au_144_Cl_60_, which is isoelectronic with Au_144_(SR)_60_. It was shown that a fully symmetric icosahedral structure is stable at charge states +2 and +4, but not at neutrality. Furthermore, other studies have experimentally illustrated charge-dependent thermal stability of Au_144_(SC_6_H_13_)^55^. All our data have been measured in the uncharged state and, therefore, we cannot comment on a charge-dependent structure in Au_144_(*p*-MBA). However, when considering our new PDF data, applicable for detailed nanostructure analysis, along with the previously published powder x-Ray diffraction (PXRD), STEM and NMR data, this again points to a scenario where the icosahedral core structure and decahedral structure both exist with very similar energies. Small differences in the electronic state of the cluster from, for example, charge or ligand binding, could lead to different structures and, possibly, even switching between the different structural forms.

As noted above, the full staple arrangement on the decahedral clusters cannot be deduced from the PDFs and X-ray scattering data must be combined with techniques sensitive to the organic ligands to establish the total structure. If considering also the experimental PDFs from the icosahedral structures (that is, with SC6 and PET ligands), we note that neither the Bahena *et al.*^22^ or Lopez-Acevedo *et al.*^20^ models fully capture the details in the PDFs, for example, in the peaks between 4 and 5 Å in Fig. 3a. Some structural details exist, which are not present in the established models. Therefore, further studies of the structure are needed, where scattering is combined with theory^54^ and spectroscopy^56^, to establish the total structure.

The polymorphism seen in our data suggests many new studies of gold nanoclusters. The close energies between different cluster structures may not be limited to the Au_144_(SR)_60_ cluster family, but exist in a larger size range and in different materials systems. The presence of polymorphism challenges some of the characterization methods that are used for structure solution. For example, when applying single-crystal XRD, the crystallization process works as a structural sieve that will favour only one cluster polymorph over others that may be present in suspension, resulting in an incomplete picture. As we show, PDF will see the average sample and any structural heterogeneity will be observed in the data. Compared with electron beams used for STEM studies, X-rays are much less perturbing of the system and any structural changes due to beam irradiation are therefore less probable. Furthermore, PDF allows to distinguish between seemingly similar clusters, that is, Au_130_(SR)_50_, Au_133_(SR)_52_ and the two forms of Au_144_(SR)_60_.

In summary, we have shown by means of total scattering PDF analysis on well-characterized samples of Au_144_(SR)_60_ that the clusters can take two distinct structures: a truncated decahedron structure (Form II) and the previously proposed icosahedral structure (Form I). The two structures have been isolated in samples with *p*-MBA and SC6 ligands, respectively, but in samples with SC4 and SC12, the two structures are seen to coexist, indicating that the energy of the two structures are very close to each other. In recent times, several new metal clusters have been isolated in the size range from 50 to 300 atoms^57^,^58^. The structures of many of these clusters remain undetermined, owing to difficulties in crystallizing the clusters into a large, single crystal suitable for structure determination. We believe that PDF will be an excellent tool for these studies and, when combined with spectroscopic methods, will be able to provide full structure solutions to many new nanomaterials.

## Methods

### Synthesis and purification of Au_144_(SR)_60_

Au_144_(*p*-MBA)_60_ was synthesized as in Ackerson *et al.*^39^ and described in Supplementary Note 7, along with the purification and characterization. Au_144_(SR)_60_, where SR=PET, S4, S6 and S12, synthesis followed Qian *et al.*^59^, also discussed further in Supplementary Note 7. The samples were characterized by ESI–MS (Supplementary Fig. 1 and Supplementary Table 1) and electrochemical methods (Supplementary Fig. 14–16).

### X-ray total scattering experiments

Total scattering data were acquired during three different beamtimes at three different facilities. For all samples, the cluster powders were loaded in Kapton tubes with inner diameter of 1 mm. Data for both samples of the Au_144_(*p*-MBA)_60_ cluster was obtained at ID11 at the European Synchrotron Radiation Facility with an X-ray wavelength of 0.1774 Å at 100 K. For the Au_144_(PET)_60_, Au_144_(SC6)_60_, Au_144_(SC4)_60_ and Au_144_(SC12)_60_ clusters, data were measured at beamline 11-ID-B at the Advanced Photon Source, at Argonne National Laboratory. Here, data were measured at 100 and 300 K with X-ray wavelength 0.143 Å. Additional data sets for the Au_144_(PET)_60_ were furthermore measured at the X7B beamline at room temperature with X-ray wavelength of 0.319 Å, as well as at the X17A beamline, X-ray wavelength 0.186 Å at 100 and 300 K, both at the National synchrotron light source facility at Brookhaven National Laboratory.

The experimental powder diffraction patterns were integrated using the programme *Fit2D*^60^ and Fourier transformed to obtain the PDF using the programme *PDFgetX3*^61^. Modelling was done using *DiffPy-CMI*. Details on the PDF analysis can be found in Supplementary Note 1.

### Data avaliability

The X-ray total scattering data that support the findings of this study are available at https://github.com/sbillinge/ncomm-goldnp-2016 with the 10.5281/zenodo.51551 (ref. 62).

## Additional information

**How to cite this article:** Jensen, K. M. Ø. *et al.* Polymorphism in magic-sized Au_144_(SR)_60_ clusters. *Nat. Commun.* 7:11859 doi: 10.1038/ncomms11859 (2016).

## Supplementary Material

Supplementary Information Supplementary Figures 1-16, Supplementary Tables 1-5, Supplementary Notes 1-7 and Supplementary References

## Figures and Tables

**Figure 1:**
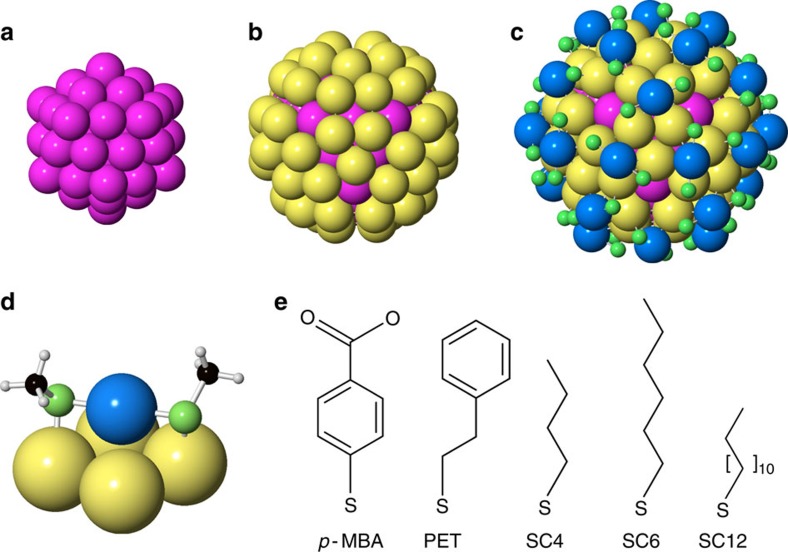
Structure of the icosahedral Au_144_(SR)_60_ cluster. (**a**) Fifty-four atom gold core consisting of two Mackay icosahedron shells. (**b**) The icosahedral gold core (pink) is covered by 60 gold atoms (yellow) making up the grand core. (**c**) Total structure, where the grand core is covered in ‘staples'—green atoms represent sulfur, whereas blue atoms represent gold in the staple structure. The organic carbon chains have been left out for clarity. (**d**) Illustration of staple structure on gold surface. (**e**) Thiolate ligands used in the study. From left: *p*-MBA, PET, SC4, SC6 and SC12.
