# Supplementary material for: Polymorphism in magic-sized Au144(SR)60 clusters
Source: Nat Commun. 2016 Jun 14;7:11859. doi: 10.1038/ncomms11859 (PMC4911633; doi:10.1038/ncomms11859)
Supplement: Supplementary Information — Supplementary Figures 1-16, Supplementary Tables 1-5, Supplementary Notes 1-7 and Supplementary References [file ncomms11859-s1.pdf]

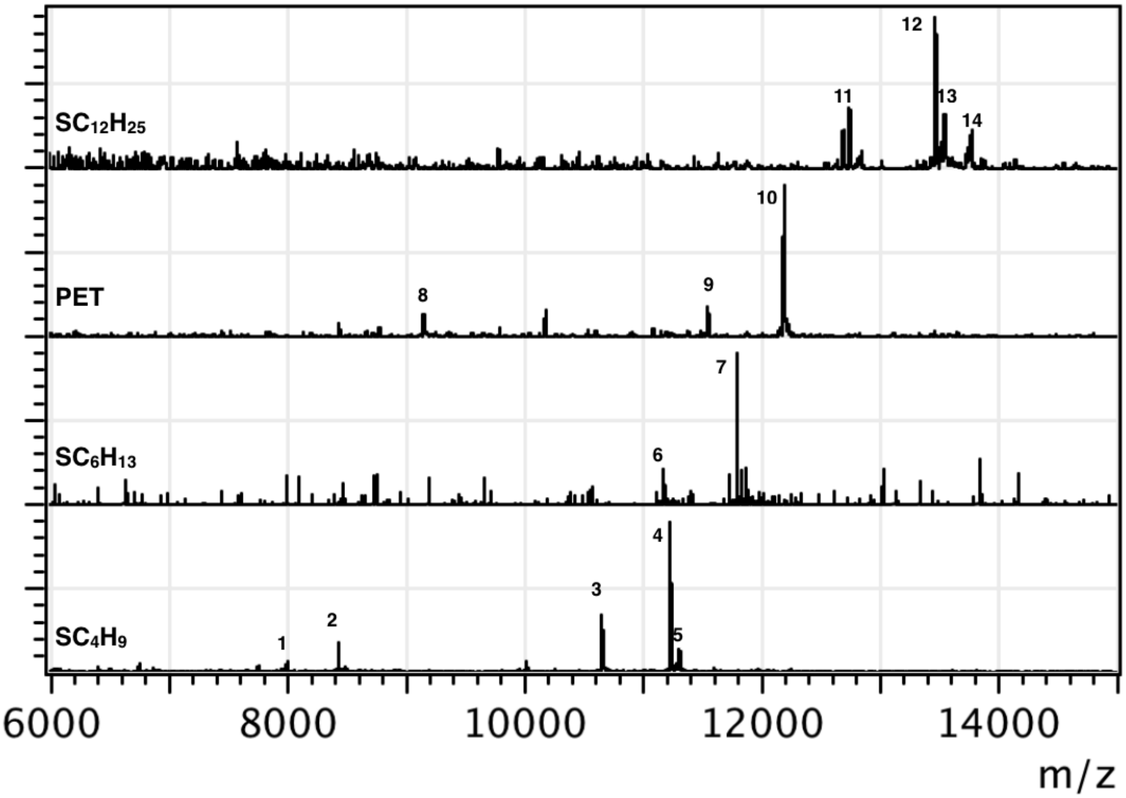

Supplementary Figure 1: ESI-MS Spectra for Au_{144} products. Peak assignments are given in Supplementary Table 1.

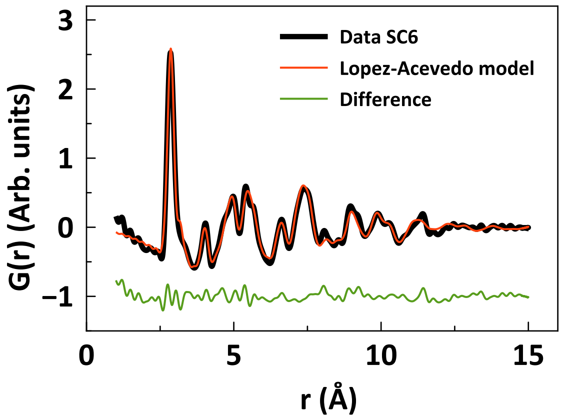

Supplementary Figure 2: Fits of the Lopez-Acevedo model to the SC6 data.

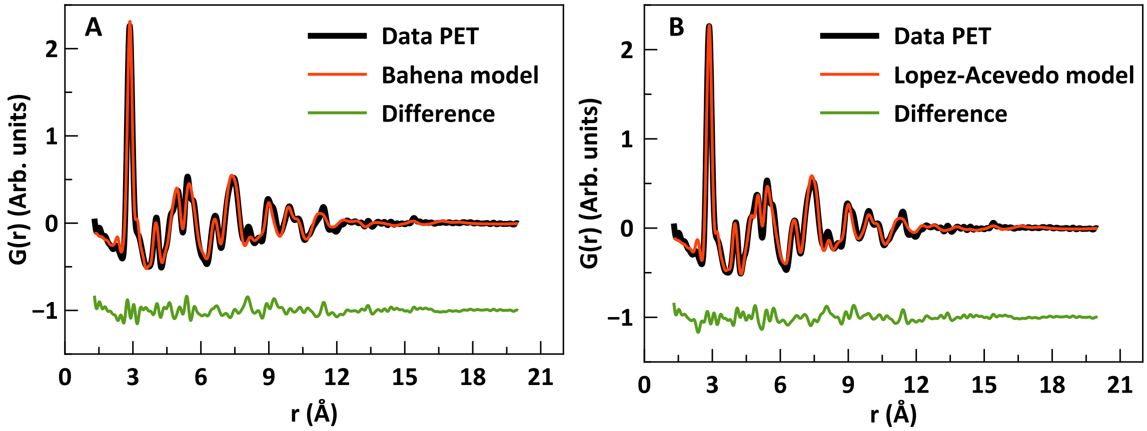

Supplementary Figure 3: Fits of the Bahena (A) and Lopez-Acevedo (B) model to the PET data.

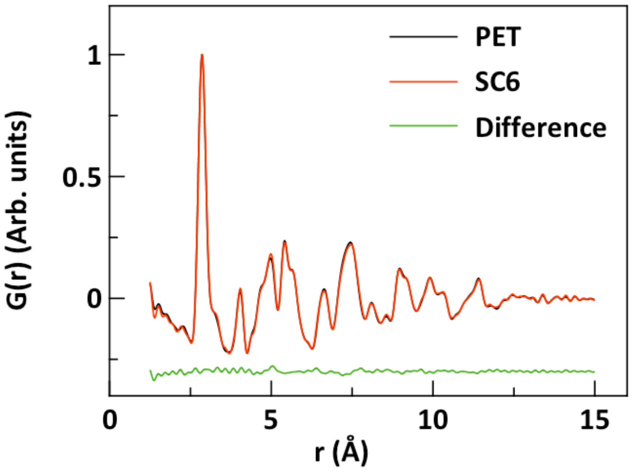

Supplementary Figure 4: Difference between the experimental PDFs obtained for PET and SC6 protected samples.

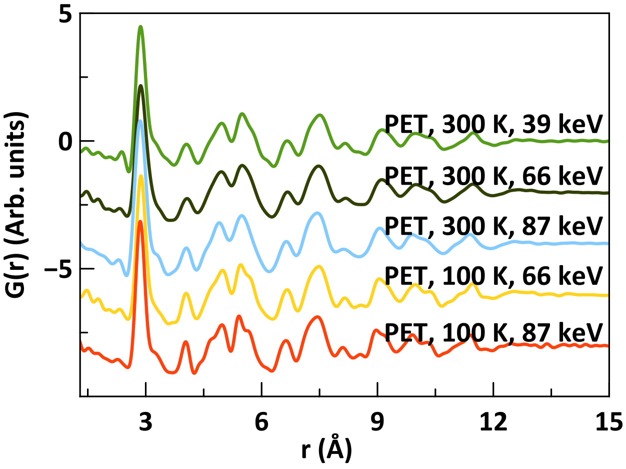

Supplementary Figure 5: Data for the $Au_{144}(PET)_{60}$ sample collected at 100K and 300K, and 39 keV, 66 keV and 87 keV.

The structural features are the same for all data sets; the only differences between the PDFs are from increased *r*-resolution with higher beam energy (allowing higher accessible Q_{max}) and peak sharpening at low temperatures.

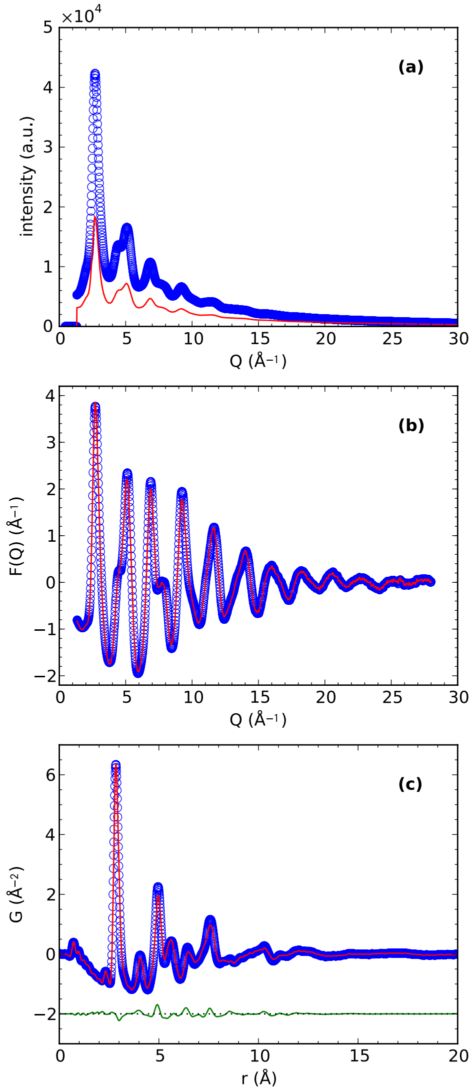

Supplementary Figure 6: X-ray PDFs measured from 2 batches of Au_{144}(*p*-MBA)$_{60}$. Sample I in blue, sample II in red. (a) Raw total scattering data. (b) Reduced scattering structure function $F(Q)$ and (c) the corresponding PDFs $G(r)$. The difference between the PDFs plotted in green yields residuum R_w = 7.8%.

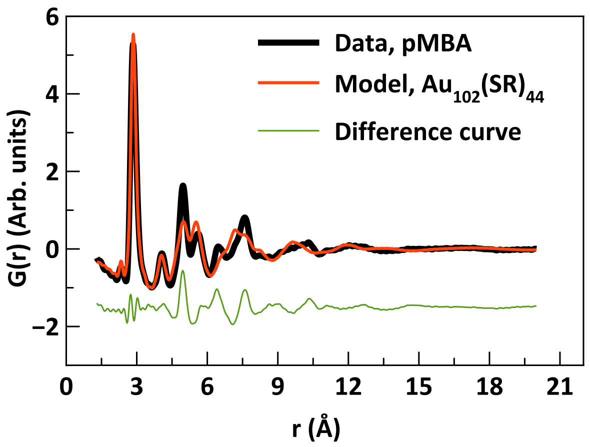

Supplementary Figure 7: Fit of the $Au_{102}SR_{(44)}$ structure to the pMBA data. R_W = 25%

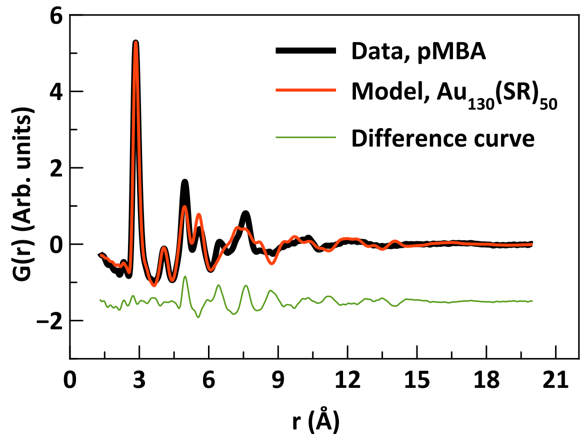

Supplementary Figure 8: Fit of the $Au_{130}SR_{(50)}$ structure to the pMBA data. R_W = 22%

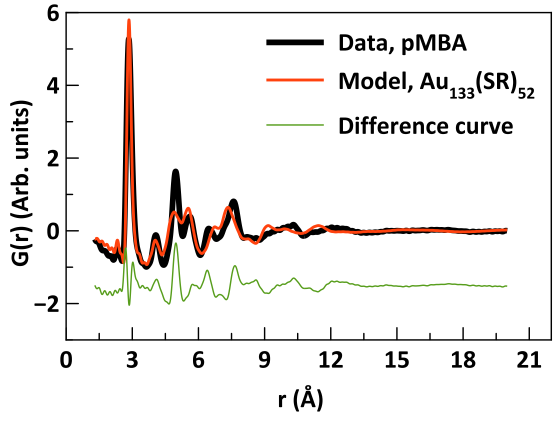

Supplementary Figure 9: Fit of the $Au_{133}SR_{(52)}$ structure to the pMBA data. R_W = 32%

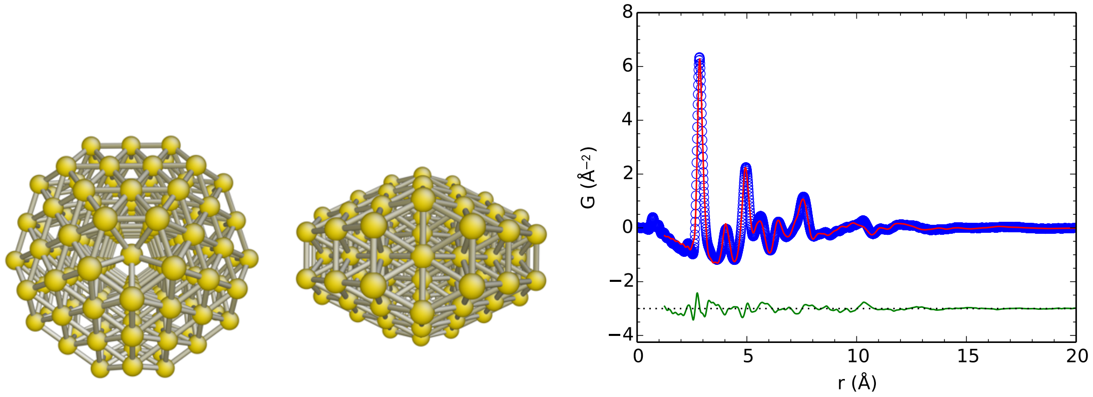

Supplementary Figure 10: Left: Top and side view of the MD6441 model, with exactly 144 gold atoms. Right: Fit of the MD6441 model to the Au_{144}(p-MBA)$_{60}$. The data are shown in blue, the model in red, and the difference curve in green.

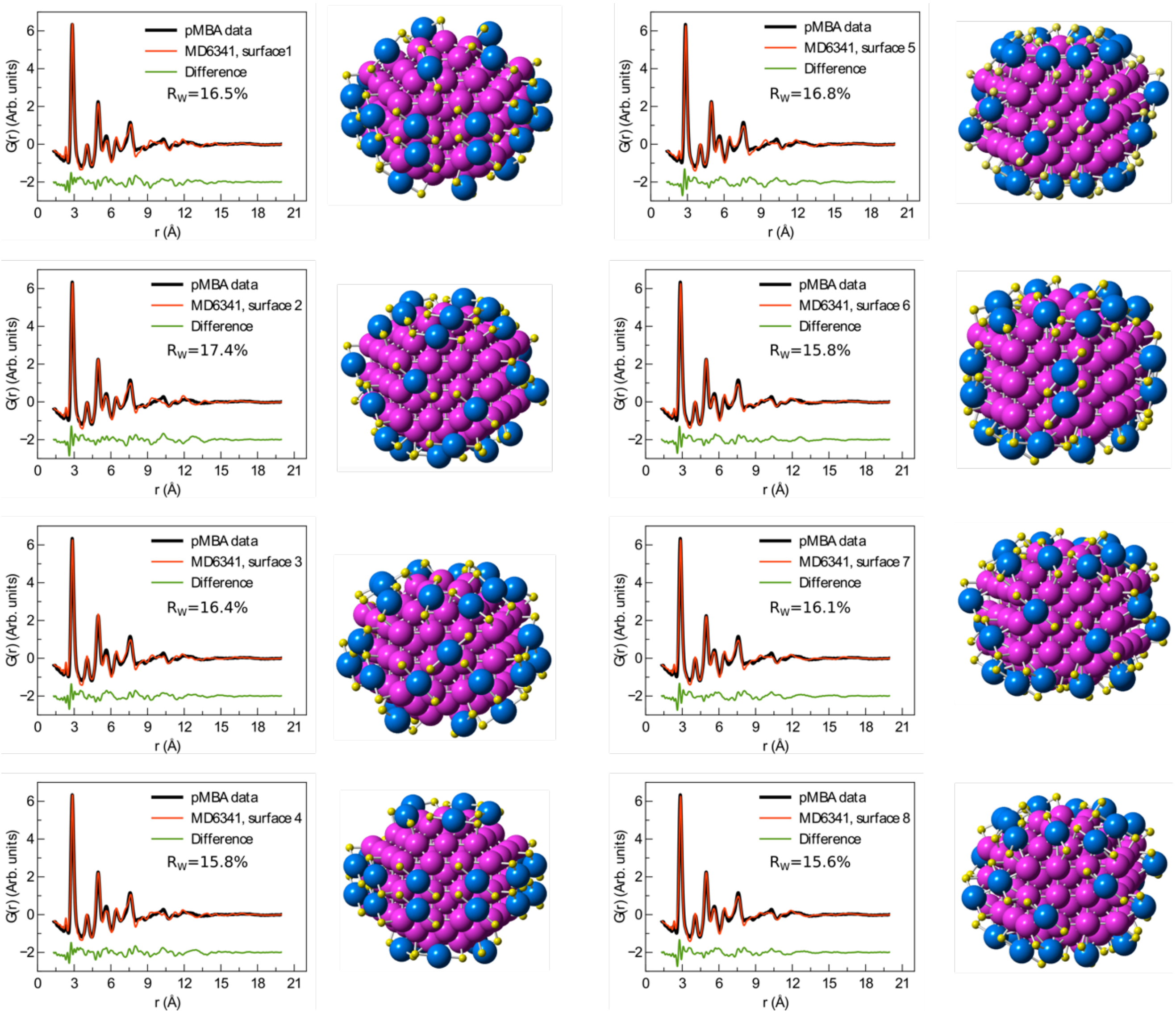

Supplementary Figure 11: Fits and structures of staple-covered MD6341. In the structural model, the MD6341 core is shown with pink atoms, while the gold bound in the staple is shown in blue. Yellow spheres show sulfur. The R_w values for each fit are given in the figure.

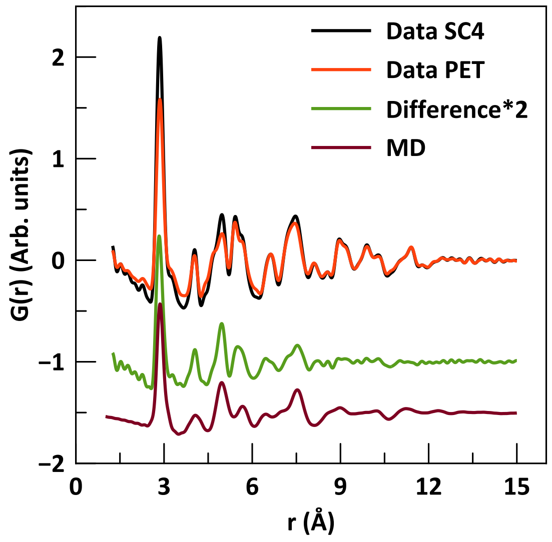

Supplementary Figure 12: Data for the $Au_{144}(PET)_{60}$ and $Au_{144}(SC4)_{60;}$ the difference between them and the calculated PDF from the 114-atom MD model. The difference curve has been doubled in intensity for clarity.

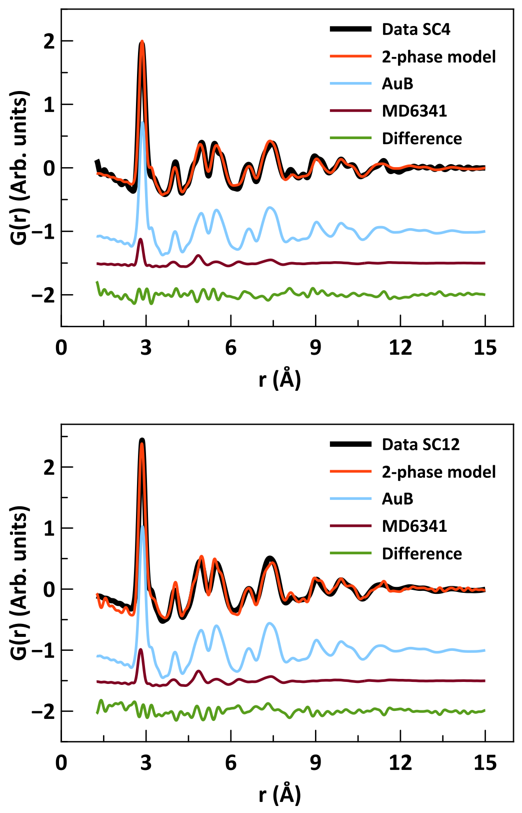

Supplementary Figure 13: Two-phase fits for $Au_{144}(SC4)_{60}$ and $Au_{144}(SC12)_{60}$ data, illustrating the presence of both icosahedra and decahedra

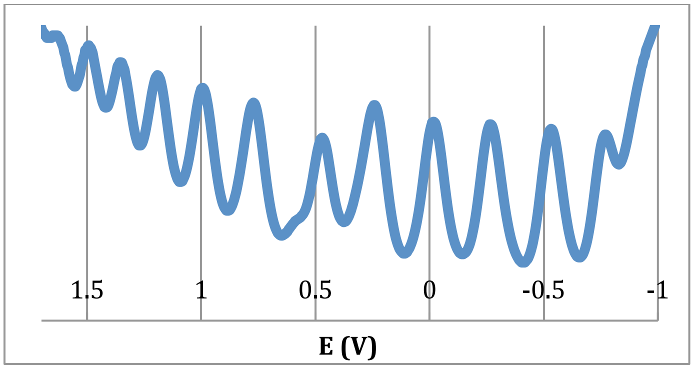

Supplementary Figure 14: DPV of $Au_{144}(SC_4)_{60}$ starting run from negative to positive potentials.

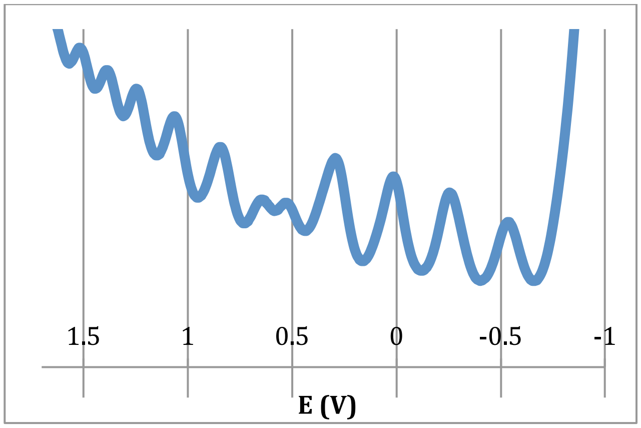

Supplementary Figure 15: DPV of $Au_{144}(SC_6)_{60}$ starting run from negative to positive potentials.

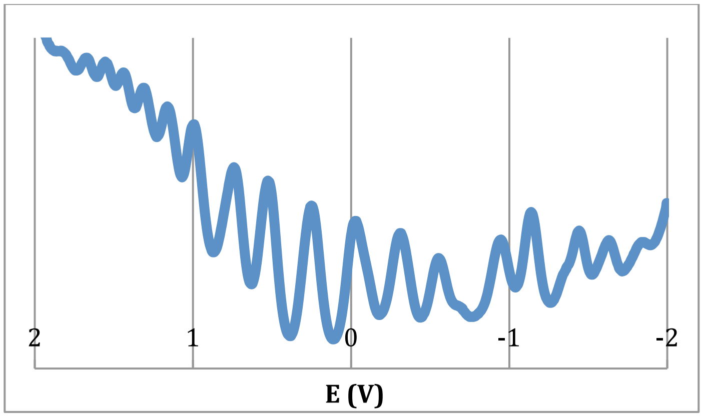

Supplementary Figure 16: DPV of $Au_{144}(PET)_{60}$ starting run from negative to positive potentials.

Supplementary table 1: Assignments of major peaks in ESI-MS spectra.

Peak index	Assignment	Calculated Mass	Observed Mass
1	$Au_{137}(SC_4H_9)_{56}^{4+}$	7993	7994
2	$Au_{144}(SC_4H_9)_{60}^{4+}$	8427	8429
3	$Au_{137}(SC_4H_9)_{55}^{3+}KNaH$	10651	10651
4	$Au_{144}(SC_4H_9)_{59}^{3+}KNaH$	11236	11227
5	$Au_{144}(SC_4H_9)_{60}^{3+}\ K_2Cs$	11306	11304
6	$Au_{137}(SC_6H_{11})_{55}^{3+}KNa_2$	11173	11173
7	$Au_{144}(SC_6H_{11})_{60}^{3+}KNa_2$	11785	11787
8	$Au_{144}(PET)_{60}^{3+}$	9147	9144
9	$Au_{137}(PET)_{55}^{3+}K_2Na$	11545	11545
10	$Au_{144}(PET)_{60}^{3+}K_2Na$	12187	12185
11	$Au_{137}(SC_{12}H_{25})_{55}^{3+}CsNaH$	12740	12739
12	$Au_{144}(SC_{12}H_{25})_{59}^{3+}SK_3$	13465	13464
13	$Au_{144}(SC_{12}H_{25})_{60}^{3+}CsNaK$	13543	13543
14	No isotope pattern		

Supplementay table 2: Results from the Bahena and Lopez-Acevedo models fitted to the $Au_{144}(PET)_{60}$ data

	Bahena	Lopez-Acevedo
R_w (%)	15.0	15.8
U_{core} (Å^2)	0.006(4)	0.004(3)
U_{shell} (Å^2)	0.02(1)	0.02(1)
δ_2 (Å)	5(1)	6(2)
ε	0.013(2)	0.015(2)

Supplementary table 3: Results from fits to the Au_{144}(*p*-MBA)$_{60}$ data

Model	R_w	$U_{iso,c}$ (Å^2)	$U_{iso,s}$ (Å^2)	δ_2 (Å)	ε[¶]	BPA
Lopez-Acevedo	0.361	0.038(6)	0.025(9)	7.85(4)	0.0171(9)	-
Bahena	0.353	0.021(2)	0.060(8)	7.42(7)	0.0153(9)	-
Au_{147} icosahedron	0.307	0.029(3)	0.021(3)	7.1(1)	-0.0019(9)	2.531
Au_{147} cuboctahedron	0.245	0.0130(9)	0.14(1)	6.3(1)	-0.0019(9)	3.020
Au_{147} *hcp*	0.259	0.012(1)	0.042(8)	5.3(2)	-0.0063(7)	2.939
Au_{141} *fcc*	0.223	0.020(1)	0.29(7)	6.3(1)	-0.0090(7)	3.149
Au_{141} *fcc* + Au_{147} *hcp**	0.184	0.0129(3)	0.09(2)	5.5(2)	-0.0075(7)	3.033
Au_{141} *fcc* + Au_{147} *hcp**[§]	0.163	0.031(3)	0.006(1)	6.8(2)	-0.029(2), -0.0070(8)	3.033
Au_{144} MD6441[§]	0.158	0.011(3)	0.0025(3)	5.0(5)	-0.018(2) 0.002(1)	2.861
Au_{114} MD6341[§]	0.163	0.010(2)	0.0022(5)	4.7(6)	0.00723(6) 0.00740(7)	3.175

[¶] *The expansion coefficient* **ε** *was obtained from the ratio of the mean bond length to the bond length in bulk gold standard.*
** The expansion coefficients in both phases were tied to the same value in the refinement as were the ADPs of their core and shell atoms. The refined hcp fraction was 55(3)% for both fit protocols.*
[§] *Fitted with separate expansion coefficients for core and shell atoms listed as (ε_c, ε_s)*
See supplementary note 4 for a description of BPA.

Supplementary table 4: Fits of the Bahena model to data from $Au_{144}(SC4)_{60}$, $Au_{144}(SC6)_{60}$ and $Au_{144}(SC12)_{60}$

Sample	R_w	$U_{iso,c}$ (Å^2)	$U_{iso,s}$ (Å^2)	δ_2 (Å)	Expansion rate
**SC4**	17.9%	0.007(5)	0.02(5)	6(2)	0.012(1)
**SC6**	16.3%	0.007(4)	0.02(2)	5(1)	0.011(1)
**SC12**	18.6%	0.008(5)	0.03(1)	6(1)	0.012(1)

Supplementary table 5: Two phase fits to data from $Au_{144}(SC4)_{60}$, $Au_{144}(SC6)_{60}$ and $Au_{144}(SC12)_{60}$. For all the two-phase refinements, the U-values (for core and shell) were constrained to take the same values for both the decahedral and icosahedral model. The δ_2 value was also constrained to one value for the two phases. The fits for SC4 and SC12 are shown in Supplementary Figure 13.

**Sample**	R_w	$U_{iso,c}$ (Å^2)	$U_{iso,s}$ (Å^2)	δ_2 (Å)	**Phase fraction, decahedra**	**Expansion, icosahedron**	**Expansion, decahedron**
**SC4**	15.7%	0.006(6)	0.02(2)	6(2)	12%	0.062(3)	-0.02(1)
**SC6**	15.8%	0.006(5)	0.02(2)	5(2)	1%	0.057(2)	0.04(1)
**SC12**	16.4%	0.007(6)	0.03(1)	6(2)	14%	0.061(3)	-0.02(1)

Supplementary Note 1: The PDF method

The PDF method is here briefly described, while more information can be found elsewhere.[1,2] The experimental PDFs are obtained from raw total scattering data using the program *PDFgetX3*[3] by subtracting background scattering as well as Compton and fluorescent intensities that do not carry structural information. The remaining coherent scattering intensity $I(Q)$ is then normalized by the atomic scattering factors to yield the total scattering structure function $S(Q)$:

$$S(Q) = \frac{I(Q)-\langle f(Q)^2\rangle}{\langle f(Q)\rangle^2} + 1 \qquad \text{(eq. 1)}$$

Here, the average values of the scattering factor $f(Q)$ are calculated over all chemical species in the specimen. The structure function can then be expressed in the form of reduced scattering structure function $F(Q) = Q(S(Q) - 1)$ which can be evaluated from an atomic structure by the Debye scattering equation:

$$F(Q) = \frac{1}{N\langle f(Q)\rangle^2} \sum_{i\neq j} f_i(Q) f_j(Q) \frac{\sin(Qr_{ij})}{r_{ij}} \qquad \text{(eq. 2)}$$

Here f_i is the scattering power of atom i and r_{ij} is the distance between atoms i and j. By applying a Fourier transformation, the sine-terms in the Debye equation transform to Dirac delta functions and thus yield real-space probabilities of interatomic distances in the material.

$$G(r) = \frac{2}{\pi}\int_{Q_{min}}^{Q_{max}} F(Q)\sin(Qr)\, dQ \qquad \text{(eq. 3)}$$

$$G(r) = \frac{1}{Nr\langle f\rangle^2} \sum_{i\neq j} f_i f_j \delta(r - r_{ij}) \qquad \text{(eq. 4)}$$

The integration limits Q_{min}, Q_{max} in the Fourier integral are the Q-limits of the data measured in the X-ray experiment.

Given a specific structure model, the expected PDF can be simulated from atom coordinates by evaluating $F(Q)$ with an extra term for pair distance uncertainty arising from atom displacements due to thermal motion or static disorder:

$$F(Q) = \frac{1}{N}\sum_{i\neq j}\frac{\exp\left(-\frac{1}{2}U_{ij}^{(c)}Q^2\right)\sin(Qr_{ij})}{r_{ij}} \qquad \text{(eq. 5)}$$

The scattering factors are here omitted for simplicity. The static and thermal atom displacements cause a spread in pair distances and thus broadening of PDF peaks, but at a short distance the peaks are sharper due to correlated motion of tightly bound atoms.[4] The U_{ij} term for the mean square deviation of the atom distance r is therefore adjusted according to the correlated motion model by Jeong *et al* as:[5]

$$U_{ij}^{(c)} = U_{ij}\left(1 - \frac{\delta_2}{r_{ij}^2}\right) \qquad \text{(eq. 6)}$$

For a given structure model, the agreement of its simulated PDF with the measured data is evaluated as a least-squares residuum R_w:

$$R_w = \sqrt{\frac{\sum_n \left(G_{obs,n} - G_{calc,n}\right)^2}{\sum_n G_{obs,n}^2}} \qquad \text{(eq. 7)}$$

The parameters of a structure model, such as atom positions or displacement parameters can be refined to minimize the difference between experimental and simulated PDFs. Here, we do this using the program suite DiffPy-CMI.

Supplementary Note 2: Cluster refinement procedures

All cluster modeling was done using the same procedure. All atomic positions in the cluster model were given as *xyz* coordinates, taken either from published models, closed packed derived models, or Mark decahedrons models. For all refinements, these positions were kept fixed which ensured that the basic structure did not change. However, the atomic displacement parameters (ADP) were refined, along with the δ_2 value taking into account the correlated motion described above. An expansion coefficient allowing the whole structure to expand isotropically was also refined, which served as a way to fit the interatomic distances without changing the structural model. For certain refinements (noted in the main text) separate ADPs and expansion factors were refined for the shell and core atoms, where the shell atoms were considered as those with 9 or fewer Au neighbors. The atoms were considered neighbors if their distance was smaller than the midpoint between the first and the second neighbor separations in a bulk *fcc* lattice. A scale factor was furthermore refined to match the PDF intensities. The PDFs were refined in the *r*-range 1-20 Å. The *Q*-range used in the Fourier transform of the calculated Debye intensities was 1.3 to 28 Å^{-1}, i.e. the same range used when obtaining the experimental PDFs from total scattering data from 11-ID-B, APS and ID11, ESRF.

Supplementary Note 3: Scattering power

The experimental PDF pattern contains contributions from the gold core as well as from the thiolate ligand molecules $(SR)_{60}$ that passivate the surface. In a PDF measurement the signal from each atom is scaled by its scattering strength, which is proportional to the number of electrons, or atomic number, therefore we can estimate the relative ratio of the gold core signal to the total PDF. The total scattered intensity is proportional to:

$$\left(\sum_i f_i\right)^2 - \sum_i f_i^2 \qquad \text{(eq. 8)}$$

For the Au_{144}(p-MBA)$_{60}$ cluster (containing 144 gold atoms, 60 sulfur atoms, 420 carbon atoms, 120 oxygen atoms and 300 hydrogen atoms), this implies that 50% of the diffracted radiation originates from the 144 gold atoms and is independent of thiolate arrangement. If including the 60 sulfur atoms, the model will account for 60% of the scattered radiation. Furthermore, the organic carbon chains are not rigid units and not

well ordered at *r*-distances larger than *ca.* 3 Å, meaning that they would not give rise to significant features in the final PDF. These two factors show that the contribution of the ligands to the signal in the measured PDF will be small.

Supplementary note 4: Fits of closed packed structures to the *p*-MBA data

A range of different structures were fitted to the experimental PDF from Au_{144}(*p*-MBA)$_{60}$. These included 147-atom cuboctahedron and icosahedron structures, as well as clusters formed by cutting spheres of approximately 144 atoms from *fcc* and *hcp* lattices. For the *fcc* lattice, the closest size was 141 atoms at the 8-th neighbor distance and for the *hcp* lattice the nearest structure had 147 atoms within the 14-th neighbor separation. Although these sizes are slightly different from the expected size of 144 atoms, the PDF simulation is quite insensitive with respect to the addition or removal of a few surface atoms and we wished to preserve the close-packed spherical particle. The next attempted model was a two-phase fit of the PDFs from cutouts from *fcc* and *hcp*. Mixing the PDFs from *fcc* and *hcp* models is a strategy to mimic stacking faults in close-packed structures.[6] The expansion coefficients in both phases were tied to the same value in the refinement, as were the ADPs of their core and shell atoms.

As an approximate measure of the surface energy we also include the number of free bonds per atom BPA, which was evaluated as the per-atom average $\langle 12 - Z_i \rangle$ with Z_i being the coordination number of atom *i*. The atoms were considered neighbors if their distance was smaller than the midpoint between the first and the second neighbor separations in a bulk *fcc* lattice.

The *fcc/hcp* mixture fits the data well, with the main features reproduced well and no significant missing peaks. R_w is also approaching a value what would be considered a good fit especially in the PDF literature, especially for nanoparticles.[7,8] However, issues are seen at larger *r*-values and the refined ADPs are very large, indicating deficiencies in the model. The expansion coefficient of the surface layer is negative, which seem unphysical. As well as the issues with the fit, these close-packed models do not explain the extreme stability and magic number of the 144 gold atom cluster.

Supplementary Note 5: Marks decahedra

The Marks decahedron size and shape are controlled by 4 integral coefficients N, M, K and T. Coefficient N is the number of atoms along the central 5-fold axis, M is the number of (002) shells, K is the number of atomic columns along the twin boundary, and T is a number of planes perpendicular to the central axis truncated from top and bottom of the decahedron.[9,10] Variations of these coefficients produced 20 clusters in the size range from 134 to 154 atoms, all of which were fitted to the data. Out of this group the 9 clusters generated with $N = 6$ showed remarkably better fit than the remaining structures. The fit results for the 144-atom variant, which agrees with the expected atomic composition, are in supplementary table 3. The fit using the Marks decahedron model (generated with N, M, K, T coefficients of (6, 4, 4, 1) and therefore labeled MD6441) is shown in supplementary figure 10 along with the structure.

Supplementary note 6: MD6341 with staples

The MD6341 structure has 10 facets with *fcc* (111) structure exposed (top and bottom) as well as 5 facets with *fcc* (100) *structure* (sides). Various configurations of 30 -SR-Au-RS- staples on the MD6341 were considered. Firstly, a model was created were the staples were only attached to the (111)-facets of the clusters, as this is the bonding motif that is most studied in gold-thiol structures. To get physical S-Au distances that fit to the experimental PDF, the staples were twisted: The Au atoms was kept fixed centrally between the two gold anchors, but the sulfur atoms were displaced from the anchors. This same motif is seen in the DFT structures, and is allowed within the degrees of freedom in the staple structure. While an acceptable fit was obtained, the proximity of the staples gives rise to some unphysical short atom-atom distances (e.g. Au-S distances of 1.44 Å). The structure also seems unlikely due to naked (100) surfaces. Models with different rearrangements of the staples were then constructed, where thiolate bonding on the (100) surfaces was included. We assumed the same staple structure, where the -SR-Au-SR- staples were anchored on the (100) surface, although this has not yet been reported on Au (100) surfaces. Several different models were constructed, of which some are presented in supplementary Figure 12. All of the models give acceptable fits to the data, with R_w values of ca. 15-16%. The PDF is somewhat sensitive to the staple attachment, as subtle

differences between the features in the fitted PDF can be observed.

Supplementary note 7: Synthesis of $Au_{144}(SR)_{60}$

Chemicals:

Butanethiol (Kodak), hexanethiol (Aldrich, 95%), dodecanethiol (Aldrich, 97%), phenylethanethiol (Aldrich 99%), *p*-MBA (TCI >95%), acetone (Omnisolv, 99.8%), methanol (Fischer, 99.9%), dichloromethane (Omnisolv, 99.96%), sodium hydroxide pellets (Fischer, ACS grade), nanopure water, sodium borohydride (Aldrich > 98%) ammonium acetate (Fisher, ACS grade)

SR = p-MBA

Synthesis proceeded by minor modifications of a previously reported protocol.[11] *para*-mercaptobenzoic acid (*p*-MBA, TCI America, 860 mg, 5.58 mmol) was dissolved in 0.3 M aqueous NaOH solution (58.61 mL, 95.2 mM) and allowed to equilibrate overnight. The following day a Au(I)-pMBA polymer was prepared by adding 225.6 mL methanol, 157.6 mL H_2O, 51.44 mL aqueous $HAuCl_4$ (32.5 mM) solution, and 45.36 mL pMBA solution to a 1L fleaker (in that order) and allowed to stir at room temperature overnight. The polymer solution was initially a slightly opaque canary yellow color, which turned clear and colorless the following day. Au_{144}(*p*MBA)$_{60}$ was then synthesized by reducing 25 mL aliquots of polymer with freshly prepared aqueous $NaBH_4$ (25 mL, 150 mM) in 50 mL conical vials over a period of 2 hours while shaking vigorously on a vortexing mixer. The particles were then precipitated with the addition of 1 mL 5 M NH_4OAc and 24 mL methanol, followed by centrifugation at 4,000 rpm for 10 min. The supernatant was discarded, the particles (which appeared dark brown/black) were washed twice with approximately 5 mL of nanopure water and precipitated again as described above. The resulting supernatant was decanted and the pellet was allowed to air dry. The particles were redispersed in water and analyzed by polyacrylamide gel electrophoresis (16% PAGE run at 110 V for 1 hour in 1X TBE). Polyacrylamide gel electrophoresis is well established for validating thiol protected nanocluster preparations.[12] After fractional precipitation with first 37.5% aqueous methanol and subsequenty 41% aqueous methanol, both in 100mM mM NH_4OAc the product was indistinguishable from known

$Au_{144}(pMBA)_{60}$ sample by PAGE. Nanocluster standards of $Au_{102}(pMBA)_{44}$, $Au_{144}(pMBA)_{60}$ and a larger cluster with an approximate nuclearity of ~Au_{200}, demonstrated the purity of the purified $Au_{144}(pMBA)_{60}$.

SR= SC_4, SC_6, SC12, PET

All four orgaonosoluble $Au_{144}(SR)_{60}$ nanocluster preparations were synthesized by suitable modifications (i.e., substitution of ligand chain length) of a previously reported protocol.[13] $Au_{144}(SR)_{60}$ was synthesized dissolving 472 mg of $HAuCl_4$ and 760 mg of tetraoctylammonium bromide in 60 ml of methanol and this solution was allowed to stir for 15 min. Next, 6.3 mmol of the desired thiol was added to the solution and allowed to stir for 15 min. During this time a separate solution containing 456 mg of $NaBH_4$ in 24 ml of ice-cold water was prepared. The solutions were than combined rapidly and stirred. After 5 hours the stirring was stopped and a black oil settled out of the solution. The supernatant was discarded and the product was precipitated out of the oil by adding methanol. The precipitate was then centrifuged and the supernatant discarded. In order to remove any remaining thiol, the precipitate was washed with methanol. $Au_{25}(SR)_{18}^{-1}$ was removed by washing the precipitate with acetone. $Au_{144}(SR)_{60}$ was extracted from the precipitate with dichloromethane and then dried. $Au_{144}(SR)_{60}$ protected by alkanethiols was further purified by size exclusion chromatography.

The synthetic products were validated by ESI-MS and voltammetry for organosoluble products, by TEM, and by polyacrylamide gel electrophoresis for water-soluble products. Supplementary Figure 1 shows the results of Electrospray Ionization Mass Spectrometry. Electrospray mass spectra were acquired on an Agilent 6220 TOF-MS using 2µl injection volumes with a flow rate of 0.3 ml/min The sheath gas temperature was set at 120 ºC and a drying gas with a flow rate of 5 L/min was used. The pressure of the nebulizer was 45 psig. A potential of 4000 V was applied for the V_{cap}. The potential applied to the fragmentor and skimmer were 75 V and 60 V, respectively. . Cs^+, Na^+, and K^+ ions were present in all samples, contributing to the appearance of multiple ion peaks. The raw data were smoothed using a moving average alorithm.

In the ESI-MS, we observe that $Au_{144}(SR)_{60}$ is the dominant product, with $Au_{137}(SR)_{56}$ detectable as a minor product. Table S5 shows the assignments of the

numbered major peaks. In addition, any of the un-numbered minor peaks we examined (40 additional peaks) we examined could also be assigned as either $Au_{144}(SR)_{60}$ or $Au_{137}(SR)_{56}$.

Differential pulse voltammetry is previously established as a means for identifying nanoclusters after purification, for instance as a column chromatographic detector.[14,15] As prepared here, $Au_{144}(SR)_{60}$ exhibits at least 14 reversible oxidation/reductions waves and the potential between each peak is almost equal for each charging event, indicating purity of material. It has been shown that the potential at which these charging events occur is related to size of the dielectric layer, solvent, and supporting electrolyte in solution.[14] The peak spacings, peak shape, and electrochemical reversibility of the system correlate to previous reports for all four of the organic soluble $Au_{144}(SR)_{60}$ compounds.[14] Differential pulse votammograms for the $Au_{144}(SC_4)_{60}$, $Au_{144}(SC_6)_{60}$ and $Au_{144}(PET)_{60}$ used in the measurements reported here are shown in Figures S 9-11. All differential pulse voltammograms were collected on a BAS 100-B potentiostat in a solution of 0.1 M tetrabutylammonium hexafluorophosphate in dichloromethane using a standard calomel electrode.

Supplementary References

1 Egami, T. & Billinge, S. *Underneath the Bragg Peaks*. 2. edn, Vol. 16 (Pergamon, 2013).

2 Farrow, C. L. & Billinge, S. J. L. Relationship between the atomic pair distribution function and small-angle scattering: implications for modeling of nanoparticles. *Acta Crystallogr. Sect. A* **65**, 232-239, (2009).

3 Juhas, P., Davis, T., Farrow, C. L. & Billinge, S. J. L. PDFgetX3: a rapid and highly automatable program for processing powder diffraction data into total scattering pair distribution functions. *J. Appl. Crystallogr.* **46**, 560-566, (2013).

4 Jeong, I. K., Heffner, R. H., Graf, M. J. & Billinge, S. J. L. Lattice dynamics and correlated atomic motion from the atomic pair distribution function. *Phys Rev B* **67**, (2003).

5 Jeong, I. K., Proffen, T., Mohiuddin-Jacobs, F. & Billinge, S. J. L. Measuring correlated atomic motion using X-ray diffraction. *J. Phys. Chem. A* **103**, 921-924, (1999).

6 Yang, X. H. *et al.* Confirmation of disordered structure of ultrasmall CdSe nanoparticles from X-ray atomic pair distribution function analysis. *Phys. Chem. Chem. Phys.* **15**, 8480-8486, (2013).

7 Masadeh, A. S. *et al.* Quantitative size-dependent structure and strain determination of CdSe nanoparticles using atomic pair distribution function analysis. *Phys Rev B* **76**, (2007).
8 Page, K., Hood, T. C., Proffen, T. & Neder, R. B. Building and refining complete nanoparticle structures with total scattering data. *J. Appl. Crystallogr.* **44**, 327-336, (2011).
9 Marks, L. D. Surface-Structure and Energetics of Multiply Twinned Particles. *Philosophical Magazine a-Physics of Condensed Matter Structure Defects and Mechanical Properties* **49**, 81-93, (1984).
10 Marks, L. D. Experimental Studies of Small-Particle Structures. *Rep. Prog. Phys.* **57**, 603-649, (1994).
11 Ackerson, C. J., Jadzinsky, P. D., Sexton, J. Z., Bushnell, D. A. & Kornberg, R. D. Synthesis and Bioconjugation of 2 and 3 nm-Diameter Gold Nanoparticles. *Bioconjugate Chem.* **21**, 214-218, (2010).
12 Kimura, K. *et al.* Size Determination of Gold Clusters by Polyacrylamide Gel Electrophoresis in a Large Cluster Region. *J. Phys. Chem. C* **113**, 14076-14082, (2009).
13 Qian, H. F. & Jin, R. C. Ambient Synthesis of Au-144(SR)(60) Nanoclusters in Methanol. *Chem. Mater.* **23**, 2209-2217, (2011).
14 Guo, R., Georganopoulou, D., Feldberg, S. W., Donkers, R. & Murray, R. W. Supporting electrolyte and solvent effects on single-electron double layer capacitance charging of hexanethiolate-coated Au-140 nanoparticles. *Anal. Chem.* **77**, 2662-2669, (2005).
15 Song, Y., Jimenez, V., McKinney, C., Donkers, R. & Murray, R. W. Estimation of size for 1-2 nm nanoparticles using an HPLC electrochemical detector of double layer charging. *Anal. Chem.* **75**, 5088-5096, (2003).
